# Hypoglycemia mediated by paraneoplastic production of Insulin like growth factor–2 from a malignant renal solitary fibrous tumor – clinical case and literature review

**DOI:** 10.1186/1472-6823-14-49

**Published:** 2014-06-17

**Authors:** Ameer Khowaja, Brianna Johnson-Rabbett, John Bantle, Amir Moheet

**Affiliations:** 1 Department of Medicine, Division of Diabetes, Endocrinology and Metabolism, University of Minnesota, 516 Delaware St SE, Minneapolis, MN 55455, USA

**Keywords:** Non islet cell tumor hypoglycemia (NICTH), Insulin like Growth Factor–2 (IGF-2), Big IGF-2, Hypoglycemia, Paraneoplastic production, Insulinoma, Solitary fibrous tumor

## Abstract

**Background:**

Hypoglycemic episodes are infrequent in individuals without a history of diabetes mellitus or bariatric surgery. When hypoglycemia does occur in such individuals, an uncommon but important diagnosis to consider is non-islet cell tumor hypoglycemia (NICTH). We report a case of NICTH associated with paraneoplastic insulin-like growth factor-2 (IGF-2) production and review current relevant medical literature.

**Case presentation:**

A 60 year old male with no relevant past medical history was referred to the endocrinology clinic with 18 month history of episodic hypoglycemic symptoms and, on one occasion was noted to have a fingerstick glucose of 36 mg/dL while having symptoms of hypoglycemia. Basic laboratory evaluation was unrevealing. Further evaluation however showed an elevated serum IGF-2 level at 2215 ng/mL (reference range 411–1248 ng/mL). Imaging demonstrated a large right suprarenal mass. A right nephrectomy with resection of the mass demonstrated a malignant solitary fibrous tumor. Post resection, the patient’s IGF-2 levels normalized and hypoglycemic symptoms resolved.

**Conclusion:**

Due to the structural and biochemical homology between IGF-2 and insulin, elevated levels of IGF-2 can result in hypoglycemia. A posttranslational precursor to IGF-2 known as “big IGF” also possesses biologic activity. Review of recent reported cases of NICTH identified widespread anatomic locations and varied pathologic diagnoses of tumors associated with paraneoplastic production of IGF-2 causing hypoglycemia. Definitive management of hypoglycemia associated with paraneoplastic production of IGF-2 consists of resection of the tumor responsible for IGF-2 production. Accumulating literature provides a firm basis for routine IGF-2 laboratory evaluation in patients presenting with spontaneous hypoglycemia with no readily apparent cause.

## Background

Hypoglycemia is a common medical problem in patients with diabetes mellitus treated with insulin or insulin secretagogues. Hypoglycemia is also associated with gastric bypass weight loss surgery [1].

Although rare, hypoglycemia is occasionally encountered in non-diabetic, non-gastric bypass patients. In such patients, hypoglycemia is usually a manifestation of pancreatic islet cell tumors producing insulin, primary or secondary adrenal insufficiency, advanced liver disease, pheochromocytoma, IGF-1 secreting tumors, hypothyroidism, substances interfering with insulin and insulin receptor mediated metabolism [non-islet cell tumor hypoglycemia (NICTH)] or antibodies interfering with insulin receptors [2].

In subjects with recurrent hypoglycemia and no history of diabetes or weight loss surgery, NICTH is an important disorder to consider in the differential diagnosis. Tumors that have been reported to cause NICTH include malignancies associated with insulin receptor antibodies, tumor necrosis factor (TNF), interleukin (IL) -1 or -6; pheochromocytoma associated with excess catecholamine production; and paraneoplastic production of IGF-1 or IGF-2 [2].

In this article, we report a case of NICTH associated with paraneoplastic IGF-2 production. We have also reviewed the current literature on the subject and describe pathophysiology, diagnostic methods and treatment options.

## Case presentation

A 60 year old male was referred to the Endocrinology clinic at the University of Minnesota for the evaluation of worsening symptoms that included diaphoresis, anxiety, inability to concentrate and episodic visual changes for the prior 18 months. Patient reported waking from sleep during the night with symptoms. He had discovered that carbohydrate rich snacks every 30 – 60 min prevented his symptoms. As a result of frequent snacking on carbohydrate containing foods, he had gained 30 pounds in the prior 12 months. He did not experience symptoms postprandially. During one of his episodes, he obtained a finger stick glucose value of 36 mg/dL. His past medical history included hypertension, dyslipidemia and obstructive sleep apnea. He did not have history of diabetes mellitus or bariatric surgery. His medications included metoprolol extended release, hydrochlorothiazide, irbesartan, amlodipine, aspirin, terazosin, simvastatin and omeprazole. He occasionally consumed alcohol and had a remote history of smoking. Physical examination was within normal limits except for body mass index (BMI) of 33.2 kg/m^2^.

**Figure 1:**
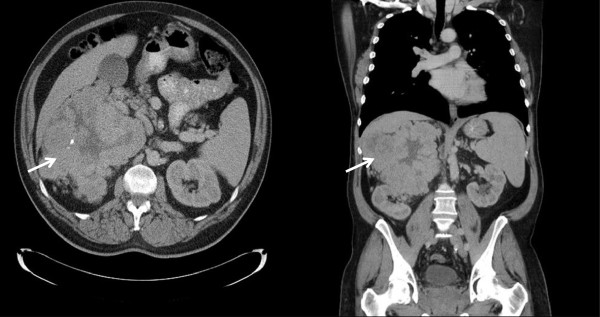
Non contrast Computed Tomography (CT) of chest, abdomen and pelvis; transverse and coronal views.

**Figure 2:**
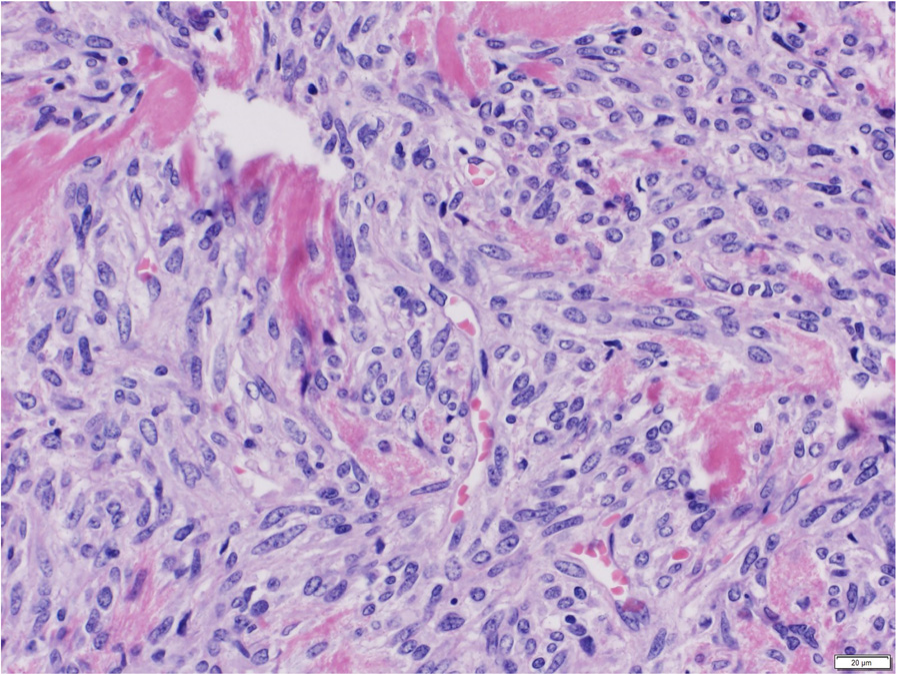
**Hematoxylin and Eosin (H&E) staining of surgically resected tumor.** Magnification 40x.

Serial finger stick blood glucose monitoring during symptomatic episodes demonstrated recurrent hypoglycemia with blood glucose values of 41, 35 and 41 mg/dL. Diagnoses considered included medication induced hypoglycemia, insulinoma, chronic liver disease, pheochromocytoma and adrenal insufficiency. These diagnoses were excluded based on laboratory evaluation (Table 1).Subsequently, IGF-2 was measured and found to be 2215 ng/mL (reference range: 414 – 1248 ng/mL). Computed Tomography (CT) without contrast of chest, abdomen and pelvis showed a large right suprarenal mass measuring 14 × 17 × 16 cm, which was lobular in shape with central necrosis and calcifications (Figure 1). Technetium radionuclide bone scan did not show any metastatic disease.The patient underwent right nephrectomy and resection of the mass. Surgical pathology showed a malignant solitary fibrous tumor with spindled to epithelioid cells and focal high-grade nuclear atypia (Figure 2). The tumor cell proliferation marker Ki67 index was elevated. The tumor was positive for CD34, CD99 and Bcl-2 consistent with a diagnosis of malignant solitary fibrous tumor.

**Table 1:** Laboratory evaluation of current case

**Test**	**Results**	**Reference range**
**Insulin (after overnight fast)**	< 2 mU/L	0 – 20 mU/L
**C-Peptide (after overnight fast)**	< 0.1 ng/mL	0.9 – 6.9 ng/mL
**Plasma glucose (after overnight fast)**	35 mg/dL	60 – 99 mg/dL
**ALT**	19 U/L	0 – 70 U/L
**AST**	27 U/L	0 – 55 U/L
**Alkaline Phosphatase**	75 U/L	40 – 150 U/L
**Total Bilirubin**	Undetectable	0.0 – 0.3 mg/dL
**Albumin**	4.4 g/dL	3.3 – 4.9 g/dL
**Total Protein**	5.2 g/dL	6.8 – 8.8 g/dL
**Hemoglobin A1c**	4.9%	4.0 – 6.0%
**α–Fetoprotein**	5.5 ug/L	0 – 8 ug/L
**IGF-I**	< 0.1 ug/L	0 – 5 ug/L
**TSH**	1.45 mU/L	0.4 – 5.0 mU/L
**Free T4**	0.98 ng/dL	0.70 – 1.85 ng/dL
**Plasma Norepinephrine**	549 pg/mL	80 – 520 pg/mL
**Plasma free Normetanephrine**	0.65 mmol/L	< 0.9 mmol/L
**Plasma Epinephrine**	117 pg/mL	10 – 200 pg/mL
**Plasma free Metanephrine**	0.23 mmol/L	< 0.5 mmol/L
**Cortisol**	11 ug/dL	4 – 22 ug/dL
**Adrenocorticophic Hormone**	23 pg/mL	< 47 pg/mL
**IGF-2 (at diagnosis)**	2215 ng/mL	414 – 1248 ng/mL

The patient’s follow-up IGF-2 levels were within the normal range (Table 2). He did not experience further episodes of hypoglycemia and was able to lose 10 lbs over the next six months. A three year follow-up CT scan of the abdomen did not demonstrate any evidence of recurrence.

**Table 2:** Post-operative serial hormone concentration

**Time post tumor resection (months)**	**Serum IGF-2 level**	**Serum IGF-1 level**
	**(288 – 736 ng/mL)**	**(81–225 ng/mL)**
**2**	562	274
**15**	670	258
**34**	582	231
**42**	563	112

## Discussion

The *IGF-2* gene is located on short arm of chromosome 11 (11p15) adjacent to the insulin (*INS*) gene [3]. IGF-2 is a 67 amino acid polypeptide with 47% sequence homology with insulin. Post translational prepro IGF-2 contains 180 amino acids with a carboxy terminal peptide of 89 amino acids and a signal peptide of 24 amino acids. Prepro IGF-2 is cleaved to form mature IGF-2 [4]. Prepro IGF-2 molecules (known as “big IGF-2”) have also been shown to have biologic activity and can produce hypoglycemia in the setting of a normal IGF-2 level [5]. It is due to the structural and biochemical homology between IGF-2 and insulin that an unregulated and elevated level of IGF-2 stimulates glucose metabolism pathways thus leading to hypoglycemia [6].

Under physiologic conditions, IGF-2 is mainly produced by the liver. However, other tissues may also produce IGF-2, allowing it to exert effects through endocrine, autocrine and paracrine pathways [7]. Physiologically, IGF-2 plays an important role in human fetal and post-natal development but whether it has significant physiological function in adults remain unknown [8]. As opposed to IGF-1, IGF-2 regulation is independent of growth hormone [9]. IGF-1 and IGF-2 both have glucose lowering potency that is approximately 5% of insulin’s potency. However, plasma concentrations of IGF-1 and IGF-2 can be 1000 times greater than insulin in NICTH allowing them to cause hypoglycemia [10]. More than 95% of IGF-2 in the circulation is bound to insulin like growth factor binding proteins (IGFBP) that have high affinity for both IGF-1 and IGF-2 [11]. In spite of structural homology with insulin, normal levels of IGF-2 do not cause hypoglycemia. At the cellular level, IGF-2 binds with IGF2R which is responsible for endocytosis, intracellular hormone transport and degradation of circulating IGF-2 [12].

Tumors producing elevated levels of circulating IGF-2 with resulting hypoglycemia are categorized as NICTH. NICTH has also been reported in conjunction with paraneoplastic production of insulin receptor antibodies, tumor necrosis factor (TNF), interleukin (IL) -1 and -6, catecholamine (pheochromocytoma) and IGF-1 [2]. For reasons not clear, there are several neoplasms noted to have high levels of IGF-2 mRNA without exhibiting elevated hormone activity [13].

The *IGF-2* gene is an imprinted gene; normally only one parental allele is expressed [14]. Loss of imprinting leads to over expression of *IGF-2* and has been demonstrated in multiple tumors including solitary fibrous tumors (independent of anatomical location), Wilm’s tumors, metastatic hemangiopericytomas, mesotheliomas, hepatocellular carcinomas, gastrointestinal stromal tumors (GIST), colorectal adenomas, osteosarcomas, rhabdomyosarcomas, leiomyosarcomas, paragangliomas, prostate cancers, breast cancers and bladder cancers [2,15]. Although these neoplasms often have elevated levels of IGF-2 mRNA, they do not always produce elevated circulating levels of IGF-2 or big IGF-2. Genetic and epigenetic mechanisms that determine isolated transcription of mRNA and the production of IGF-2 and big IGF-2 are not well understood; however, these mechanisms are thought to play an important role in carcinogenesis and tumor growth [13,16].

In a previous extensive review of this topic by de Groot and coauthors in 2007, authors noted that 41% of the IGF-2 producing tumors causing hypoglycemia were of mesenchymal origin, 43% of epithelial origin, 1% of neuroendocrine and hematopoietic origin and 14% of unknown origin [2]. Although symptoms of hypoglycemia were predominant, other symptoms included skin tags, acne and rhinophyma.

We reviewed case reports of NICTH associated with paraneoplastic production of IGF-2 from 2008 to 2012 (Table 3) [17-38]. On our review of literature, we identified 22 published reports of NICTH with elevated IGF-2 levels causing hypoglycemia. Age range of the patients was 27 – 83 years (mean age 58 years), with 14 males and 8 females. Predominant symptoms included those of hypoglycemia (diaphoresis, tremor, anxiety, loss of consciousness) and mechanical symptoms depending on site of the tumor. Some tumors were associated with acromegaly. The anatomic location of tumors in these case reports included liver (n = 9), pleural cavities (n = 5), lungs (n = 3), retroperitoneum (n = 3), bones (n = 2), pelvis (n = 2), breasts (n = 2), cranium (n = 1), kidney (n = 1), uterus (n = 1), spleen (n = 1) and adrenal gland (n = 1). The tumor involved more than one organ system in 6 patients. Fifteen patients were diagnosed with NICTH for the first time, whereas 7 patients presented with recurrent disease. Pathologic diagnosis included solitary fibrous tumor (n = 10), hemangiopericytoma (n = 3), phyllodes tumor and sarcoma (n = 2), hepatocellular carcinoma (n = 2), uterine leiomyoma (n = 1), gastric adenocarcinoma (n = 1), ovarian germ cell tumor (n = 1), pheochromocytoma (n = 1) and desmoplastic small round cell tumor (n = 1). Hypoglycemia associated with IGF-2 production has also been reported in pancreatic islet cell tumor [39]. Biochemical profiles of the patients varied and included elevated IGF-2, elevated big IGF-2 with normal IGF-2, and elevated IGF-2 to IGF-1 ratio.

**Table 3:** Cases of hypoglycemia from NICTH secreting IGF-2 reported from 2008 till 2012

**Reference**	**Age/sex**	**Tumor site**	**Tumor pathology**	**Hormone elevated**	**Management**
[17]	28, male	Retroperitoneal pelvic region	Malignant solitary fibrous tumor	IGF-2	Surgical resection of turmor
[18]	68, male	Liver	Solitary fibrous tumor	Big IGF-2	Partial hepatic resection
[19]	59, male	Lungs and bones	Meningeal hemangiopericytoma	IGF-II (elevated IGF-2/IGF-1 levels)	Interferon alpha
[20]	75, male	Left pleural cavity	Pleural solitary fibrous tumor	Big IGF-2	Surgical resection
[21]	65, male	Retroperitoneal tumor	Retroperitoneal solitary fibrous tumor	IGF-2, Big IGF-2	Surgical resection
[22]	66, female	Right inferior thorax	Solitary fibrous tumor	IGF-2	Surgical resection
[23]	53, male	Liver	Metastatic hemangiopericytoma	Elevated IGF 2/IGF-1 ratio	Right hepatectomy
[24]	64, female	Right lower thorax	Malignant solitary fibrous tumor	Elevated IGFBP	Surgical resection, radiation post operatively
[25]	83, male	Retroperitoneum	Malignant solitary fibrous tumor	Big IGF-2	Surgical resection
[26]	43, male	Right posterior cranial fossa, metastatic lesions in bilateral kidney, right iliopsoas muscle, right iliac body, thoracic vertebra 10 body and segment IV of the liver	Meningeal hemangiopericytoma	Elevated IGF-2/IGF-1 ratio	Adriamycin
[27]	59, female	Right hemithorax	Solitary fibrous tumor	Elevated IGF-2/IGF-1 ratio	Surgical resection
[28]	67, male	Pelvis	Solitary fibrous tumor	Elevated IGF-2/IGF-1 ratio	Tumor embolization and radiotherapy
[29]	49, female	Right breast	Benign Phyllodes tumor	Big IGF-2	Mastectomy
[30]	49, female	Left breast	High grade Phyllodes sarcoma	Big IGF-2	Mastectomy
[31]	80, female	Uterus	Uterine leiomyoma	IGF-II, elevated IGF-2/IGF-1 ratio	Exploratory laparotomy
[32]	69, male	Spleen, lungs, liver	Hepatocellular carcinoma	Elevated IGF-2 mRNA expression	
[33]	61, male	Liver	Poorly differentiated gastric adenocarcinoma	Big IGF-2, elevated IGF-2/IGF-1 ratio	Gastrectomy
[34]	27, female	Lung and liver	Ovarian germ cell tumor	Big IGF-2	Dexamethasone, recombinant Growth hormone
[35]	53, female	Right adrenal gland, liver		Elevated IGF-2/IGF-1 ratio	Phenoxybenzamine
[36]	45, Male	Pelvis and peritoneum	Desmoplastic small round cell tumor	Elvated IGF-2 and IGF-2/IGF-1 ratio	chemotherapy
[37]	77, male	Liver and lungs	Hepatocellular carcinoma	Big IGF-2	chemotherapy
[38]	41, male	Pleura and posterior mediastinum	Solitary fibrous tumor	Big IGF-2	chemotherapy

Currently, different assays are being used in clinical and research settings for measurement of IGF-2. These assays utilize liquid chromatography–mass spectrometry (LC-MS) [40] or immunoassay (ELISA and RIA). Western blot is used for measurement big IGF-2 [41]. The possibility of NICTH secondary to IGF-2 is suggested by low blood glucose along with suppressed insulin, C-peptide and IGF-1 levels. Concurrent normal to high morning cortisol and normal response on cosyntropin stimulation can rule out adrenal insufficiency and suggest NICTH secondary to IGF-2. IGF-2 levels are frequently within the normal range in NICTH, and assays for big IGF-2 are not commercially available. Thus, the IGF-2: IGF-1 ratio is used as a surrogate marker for big IGF-2 concentration. A ratio of >10 is considered to be clinically significant [42].

Management of NICTH can be divided into issues related to 1) hypoglycemia and 2) the underlying tumor. Hypoglycemia can be managed in a typical manner by administration of oral glucose, intravenous dextrose or glucagon depending upon the severity. Glucocorticoid therapy has been shown to suppress big IGF-2 in a dose dependent manner using doses of 30–60 mg/d of prednisolone or 4 mg/d of dexamethasone [43]. Surgical resection of the tumor, whenever possible, is the treatment of choice and, if successful, usually results in the resolution of hypoglycemia and other metabolic abnormalities. In cases where complete resection is not feasible due to tumor infiltration and/or distant metastasis, surgical debulking along with chemoradiation can be considered for treatment of hypoglycemia. The choice of chemotherapy depends upon the primary pathology of the tumor. Metastatic disease and tumor recurrence are associated with a poor prognosis.

## Conclusion

NICTH is a rare cause of hypoglycemia in the general population. It is associated with elevated levels of IGF-2, high molecular weight IGF-2 (big-IGF-2) or an increased IGF-2 to IGF-1 ratio. Diagnosis of paraneoplastic IGF-2 induced hypoglycemia is an important and time sensitive consideration since earlier detection of tumors provides a better opportunity for complete resection and subsequent resolution of hypoglycemia. Whether IGF-2 level should be routinely measured in the evaluation of hypoglycemia depends on the prevalence of the condition. Accumulating literature supports screening for IGF-2 in non-diabetic individuals who present with hypoglycemia along with suppressed insulin and c-peptide levels.

## Consent

Written informed consent was obtained from the patient for publication of this case report and accompanying images. A copy of the written consent is available for review.

## Abbreviations

IGF-1: Insulin like growth factor–1; IGF-2: Insulin like growth factor–2; NICTH: Non-islet cell tumor hypoglycemia; TNF: Tumor necrosis factor; IL: Interleukin; BMI: Body mass index; CT: Computed tomography; LC-MS: Liquid chromatography–mass spectrometry.

## Competing interest

The authors declare that they have no competing interests.

## Authors’ contribution

AK reviewed the literature and wrote the manuscript. BJR edited the manuscript, wrote the abstract and participated in the literature review. JB was involved in clinical management and reviewed and edited the manuscript. AM was involved in clinical management and reviewed and edited the manuscript. All authors read and approved the final manuscript.

## Pre-publication history

The pre-publication history for this paper can be accessed here:

http://www.biomedcentral.com/1472-6823/14/49/prepub
